# Appointments in the Paris Hospitals

**Published:** 1861-03

**Authors:** 


APPOINTMENTS IN THE PARIS HOSPITALS.—In consequence of the death of M. Després, and the retirement of MM. Ricord and Guersant, the following new appointments have been made: M. Giraldes, surgeon to the Children's Hospital; M. Follin, surgeon to the Salpetrière; M. Depaul, surgeon to the Foundling Hospital; and M. Broca, surgeon to the Bicêtre.

